# Microarray Analysis of Response of *Salmonella *during Infection of HLA-B27- Transfected Human Macrophage-Like U937 Cells

**DOI:** 10.1186/1471-2164-11-456

**Published:** 2010-07-30

**Authors:** Shichao Ge, Vittoria Danino, Qiushui He, Jay CD Hinton, Kaisa Granfors

**Affiliations:** 1 Department of Infectious Disease Surveillance and Control, National Institute for Health and Welfare, Turku, Finland; 2 Molecular Microbiology Group, Institute of Food Research, Colney, Norwich, UK

## Abstract

**Background:**

Human leukocyte antigen (HLA)-B27 is strongly associated with the development of reactive arthritis (ReA) in humans after salmonellosis. Human monocytic U937 cells transfected with HLA-B27 are less able to eliminate intracellular *Salmonella enterica *serovar Enteritidis than those transfected with control HLA antigens (e.g. HLA-A2). To investigate further the mechanisms by which HLA-B27-transfected cells allow increased replication of these bacteria, a DNA-based microarray was used for comparative genomic analysis of *S*. Enteritidis grown in HLA-B27- or HLA-A2-transfected cells. The microarray consisted of 5080 oligonucleotides from different serovars of *Salmonella *including *S*. Enteritidis PT4-specific genes. Bacterial RNA was isolated from the infected HLA-B27- or HLA-A2-transfected cells, reverse-transcribed to cDNA, and hybridized with the oligonucleotides on the microarrays. Some microarray results were confirmed by RT-PCR.

**Results:**

When gene expression was compared between *Salmonella *grown in HLA-B27 cells and in HLA-A2 cells, 118 of the 4610 *S*. Enteritidis-related genes differed in expression at 8 h after infection, but no significant difference was detectable at 2 h after infection. These differentially expressed genes are mainly involved in *Salmonella *virulence, DNA replication, energy conversion and metabolism, and uptake and metabolism of nutrient substances, etc. The difference suggests HLA-B27-dependent modulation of *Salmonella *gene expression, resulting in increased *Salmonella *replication in HLA-B27-positive cells. Among the up-regulated genes were those located in *Salmonella *pathogenicity island (SPI)-2, which play a central role in intracellular survival and replication of *Salmonella*.

**Conclusions:**

This is the first report to show the regulation of *Salmonella *gene expression by HLA-B27 during infection of host cells. This regulation probably leads to increased *Salmonella *survival and replication in HLA-B27-positive cells. SPI-2 genes seem to contribute significantly to the increased replication.

## Background

The clinical outcomes of non-typhoidal salmonellosis range from self-limiting gastroenteritis to life-threatening systemic infections [1]. Many serovars of *Salmonella enterica *cause these infections, serovar Enteritidis being among the most common [2,3]. The acute gastrointestinal infection caused by *Salmonella *may result in complications such as reactive arthritis (ReA) [4-6]. Originally, ReA was described as an aseptic inflammation that develops after an infection elsewhere in the body [7]. ReA is an asymmetric polyarthritis and the outcome of the disease ranges from mild symptoms to severe and chronic clinical manifestations. Up to 80% of patients with ReA express the HLA-B27 antigen [8,9].

Macrophages are important in the pathogenesis of *Salmonella *infections. They are an integral part of the immune response as they present antigens to the innate defence system and communicate with the adaptive immune system to resist the bacterial infection [10-12]. However, unlike many other pathogens, *Salmonella *can survive inside macrophages by adapting to this particular intracellular environmental niche. After *Salmonella *uptake into macrophages, the intracellular bacteria reside in large membrane-bound phagosomes, called spacious phagosomes (SP), which develop into *Salmonella*-containing vacuoles (SCV) [13]. The formation of SP or SCV favours the survival and replication of *Salmonella *in macrophages. Even so, *Salmonella *encounter intracellular host defence mechanisms, including reactive oxygen and nitrogen species (ROS and RNS), antimicrobial peptides, lysosomal enzymes, and adaptive immune responses [10,12]. In order to survive in the host and to avoid clearance by the host immune system, *Salmonella *express virulence factors to deal with this stressful environment [14].

Many virulence genes of pathogenic bacteria are located in large multigene chromosome regions termed pathogenicity islands (PAIs) [15]. In *Salmonella*, they are called *Salmonella *pathogenicity islands (SPIs) [16]. Two SPIs, SPI-1 and SPI-2, encode structurally similar but functionally distinct type III secretion systems (T3SS), specialized protein export machineries that *Salmonella *uses to deliver virulence proteins into the cytosol of host cells [17]. The SPI-1-encoded T3SS is active extracellularly. SPI-1 mediates invasion into non-phagocytic cells [18], and it is required for the intestinal inflammatory responses [19]. The SPI-2 virulence genes are expressed intracellularly and are required for the survival of bacteria in macrophages and systemic infections. SPI-2 mutant strains are dramatically attenuated, showing a 10^4^-fold reduction in virulence in LD_50 _in the murine salmonellosis model [16] and impaired intracellular replication and survival in macrophages [20,21].

HLA-B27 confers a very strong genetic predisposition towards the development of a group of rheumatic disorders called spondyloarthropathies (SpA), including ankylosing spondylitis (AS) and ReA. HLA-B27-positive individuals have a five-fold higher incidence of ReA than the general population [22,23]. ReA occurs following certain infections, e.g. those caused by *Salmonella *and *Yersinia *pathogens [7,8,24,25]. Expression of HLA-B27 also increases the risk that the patient will suffer a more severe and prolonged disorder [6,8]. The interaction between ReA-triggering bacteria and HLA-B27-positive subjects is abnormal and leads to increased persistence of the causative microbes/microbial antigens in HLA-B27-positive patients [26-28]. The interaction between HLA-B27 molecules and arthritogenic microbes was investigated more thoroughly using *in vitro *infected cells. Experiments investigating the invasion of HLA-B27 cells by Gram-negative bacteria, including *Salmonella*, are inconclusive. Studies have shown either decreased [29] or similar [30] levels of invasion of HLA-B27-transfected murine L fibroblasts compared with control L cell lines (L cells transfected with other MHC class I genes) or increased invasion of HLA-B27-transfected intestinal epithelial Henle-407 cells by *Salmonella *[31]. Once inside the host cells, *Salmonella *is able to replicate more quickly [32,33], and is eliminated more slowly, in HLA-B27-positive cells [30,34] than transfected control cells. The survival and persistence of *Salmonella *in the intracellular environment is associated with bacterial gene expression [35]. However, little is known about *Salmonella *gene expression in association with HLA-B27 during bacterial infection and persistence.

## Results and Discussion

### Cell surface expression of HLA-B27 and HLA-A2 molecules

The transfected HLA-B27 and HLA-A2 were always expressed on the surface of the respective cells, as detected by immunofluorescence in new batches of the cell lines (data not shown). The level of expression of the transfected molecules in U937 cells was similar to that of HLA-B51, one of the MHC class I molecules endogenously expressed by U937 cells [34]. In addition, the surface expression levels of the transfected molecules corresponded to the levels of those molecules endogenously expressed on peripheral blood monocytes [34].

### Increased replication of *Salmonella *in HLA-B27-positive U937 cells

**Figure 1:**
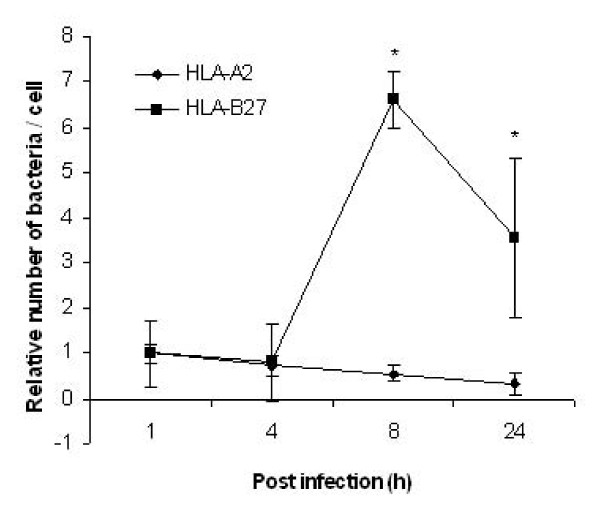
**Relative number of intracellular bacteria in each living U937 cell**. The cultured HLA-B27 or HLA-A2-transfected U937 cells were infected with complement-opsonized *S*. Enteritidis PT4 KS8822/88 and the incubation was continued at 37°C for 1, 4, 8 or 24 h after infection. At the indicated time points, the host cells were lysed and the number of bacteria per cell was counted and reported as colony forming units (CFU). For both cell lines, the number of intracellular bacteria at 1 h after infection was designated 1. The numbers of intracellular bacteria at the following indicated time points are shown as fold changes relative to the number at 1 h after infection. Values are mean ± standard deviation of duplicate samples from a representative experiment out of three with similar results. * *P *< 0.05 versus *S*. Enteritidis-infected HLA-A2 transfectants. Data were compared using Student's paired 2-tailed *t*-test.

We used the *in vitro *model of infection established earlier in our laboratory to monitor growth of *S*. Enteritidis in macrophage-like U937 cells [32-34]. Cells transfected with HLA-B27 or HLA-A2 were infected with complement-opsonized salmonellae. HLA-A2 transfected cells were used as a negative control since it is a common tissue antigen that is not related to the development of ReA [34]. At 1, 4, 8, and 24 h post infection, the host cells were lysed and the number of living intracellular bacteria per cell was determined by counting the number of colony forming units (CFU) (Figure. 1). Consistent with our previous results, no difference in the uptake of *Salmonella *was observed between the two cell lines (1 h post infection) [34]. However, more bacteria were recovered from HLA-B27-expressing cells than HLA-A2-expressing cells at 8 h and even up to 24 h post infection, suggesting that HLA-B27-transfected cells become permissive for intracellular *Salmonella *survival and replication, which is also consistent with our previous studies [32-34].

### Global gene expression profiles of *S*. Enteritidis in HLA-B27- and HLA-A2-transfected U937 cells

**Figure 2:**
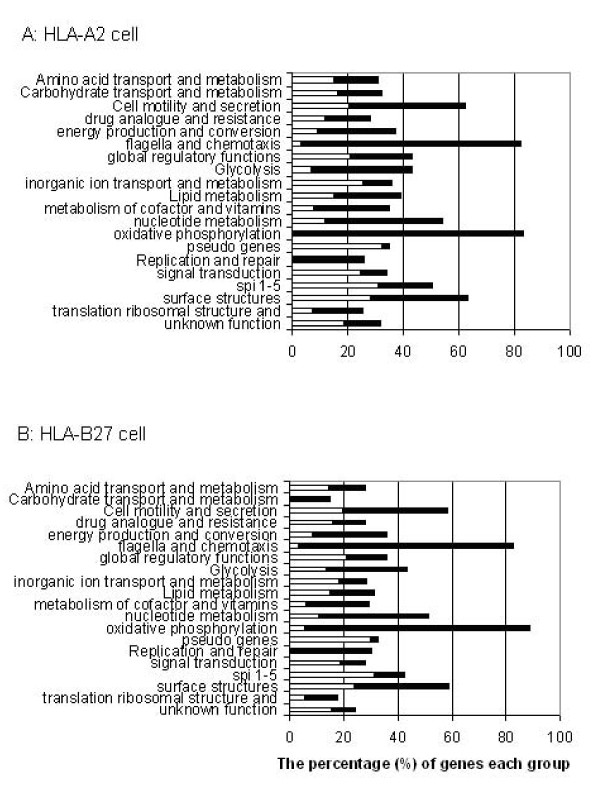
**Differential expression of *S*. Enteritidis genes divided into functional groups at 2 h time point**. Bars indicate percentages of genes in each group that showed significant changes in expression in HLA-A2 cells (A) and HLA-B27 cells (B), which were compared to cells grown in LB. The white bars indicate the proportion of up-regulated genes and the black bars the percentage of down-regulated genes for each group.

The 'SALSA' genomic *Salmonella *serovar microarray containing 5080 *Salmonella *oligonucleotides was used to monitor gene expression in *Salmonella *grown in the host cells [36]. Gene expression was compared between *Salmonella *grown in U937 cells and in LB broth. In total, 1,388 genes of *Salmonella *grown in HLA-B27 cells and 1,049 genes of *Salmonella *grown in HLA-A2 cells showed statistically significant differences in expression at 2 h post infection when compared to the transcriptome in LB broth culture. At 8 h post infection, the expression of 1,352 genes of *Salmonella *grown in HLA-B27 cells and of 1,559 genes of *Salmonella *grown in HLA-A2 cells showed statistically significant differences from *Salmonella *grown in LB broth. The genes that showed significant expression differences and were up- or down-regulated more than two-fold for each individual comparison are listed in Additional file 1: Supplemental Tables 1-6. The categories of *Salmonella *genes showing differential expression between HLA-B27 cells and LB and between HLA-A2 cells and LB at 2 h post infection can be found in Figure. 2. In particular, the functional categories analysis showed large differences in the expression of genes required for cell motility and secretion, flagellae and chemotaxis and oxidative phosphorylation. These differences in gene expression reflect bacterial adaptation to the intracellular environments.

**Figure 3:**
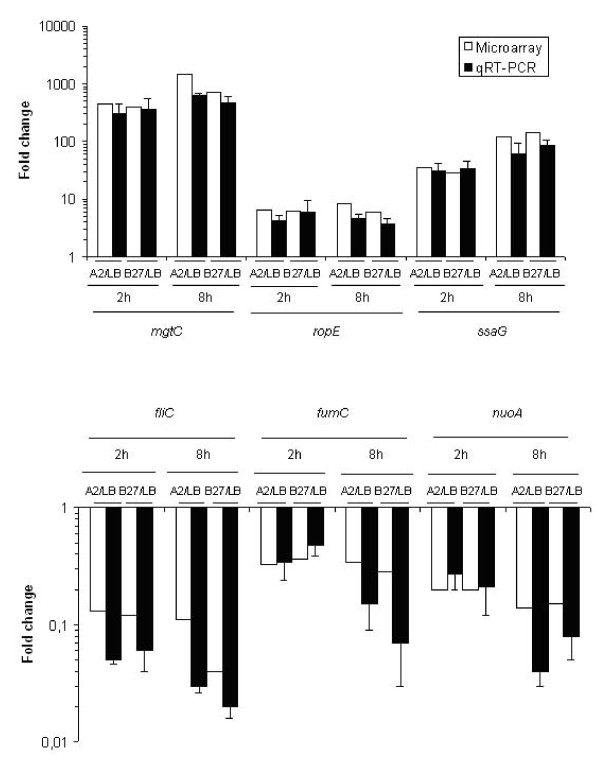
**Validation of microarray results by quantitative real time RT-PCR**. The fold changes in gene expression are shown when *S*. Enteritidis growth in HLA-A2 or HLA-B27 cells was compared to growth in LB broth. The expression levels of selected up-regulated genes (A) or down-regulated genes (B) are shown in logarithmic scale. The experiments were repeated at least three times with similar results. Values are mean numbers ± standard deviations generated from three independent experiments. Correlation coefficients (R) for comparisons of two techniques are: up-regulated genes; 0.977 (A2/LB) and 0.959 (B27/LB), down-regulated genes 0.530 (A2/LB) and 0.620 (B27/LB) (see Additional file 2: Supplemental Table S7).

To confirm the microarray data, quantitative real time RT-PCR was performed on six genes selected from different functional categories and showing differential up- or down-regulated expression between HLA-B27 and A2 cells: *mgtC*, *rpoE *and *ssaG *(up-regulated); *fliC*, *fumC *and *nuoA *(down-regulated) (Figure. 3). RT-PCR was performed using the same bacterial RNA from HLA-B27 cells, A2 cells and LB broth, as was used in microarray experiments. It is important that the genes selected covered the whole scale of the microarray data from high expression level to medium and to low expression level, as well as in differential expression from high fold changes to low fold changes in U937 cells vs. in LB, among up-regulated and down-regulated genes, respectively. The quantity of cDNA for each gene was obtained after normalization to the levels of *rfaH *cDNA, which was chosen as a control since *rfaH *is expressed stably at moderate levels under most of the conditions tested (see Additional file 2: Supplemental Table S8). The RT-PCR results of the six genes studied were consistent with the microarray data (Figure. 3), but due to the small number of genes studied with RT-PCR, data may be considered preliminary.

### *Salmonella *gene expression is regulated by HLA-B27 at the 8 h time point

**Figure 4:**
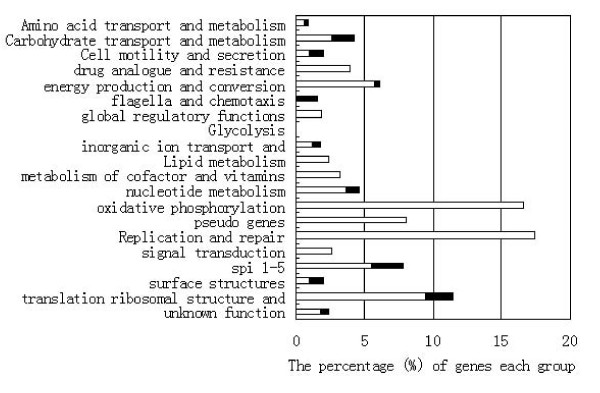
**Differential expression of *S*. Enteritidis genes between HLA-B27 and HLA-A2 cells divided into functional groups at 8 h time point**. Bars indicate percentages of genes in each group that showed significant changes in *S*. Enteritidis gene expression in HLA-B27 cells compared to in HLA-A2 cells. The white bars indicate the proportion of up-regulated genes and the black bars the percentage of down-regulated genes for each group.

To determine whether bacterial gene expression plays a role in *Salmonella *replication/persistence in HLA-B27 cells, we assessed the transcriptome of *S*. Enteritidis in the two cell lines. Differential expression of the genes betweeen HLA-B27 cells and HLA-A2 cells is based on the comparison of the gene expression in HLA-B27 cells to LB and the gene expression in HLA-A2 cells to LB. For example, for the gene whose expression is 6-fold in HLA-B27 cells compared to LB, and 2-fold in HLA-A2 cells compared to LB, the expression is 3-fold in HLA-B27 cells compared to HLA-A2 cells. No genes showed significant differences when the transcriptomes of *Salmonella *grown in HLA-B27- and HLA-A2-transfected cell lines were compared at 2 h post infection. This is consistent with the similarity observed in the intracellular growth of *Salmonella *at this time point. In contrast, 118 genes showed significant differences in expression between *Salmonella *grown in HLA-B27 cells and in HLA-A2 cells at 8 h post infection (86 genes were up-regulated and 32 genes down-regulated (see Additional file 1: Supplemental Tables S6-1 and -2, Figure 4 and Table 1). Bacterial gene expression reflects the environmental conditions in which the bacteria reside. The difference between bacteria grown in HLA-B27 and HLA-A2 cells suggests that HLA-B27 modulates the intracellular environment and causes changes in *Salmonella *gene expression during macrophage infection, resulting in increased survival and replication in HLA-B27 cells. This modulation may be due to changes in host cell signalling pathways [33,37]. This is the first observation showing that *Salmonella *gene expression is influenced by HLA-B27. The analysis of functional categories revealed that the groups of genes most altered in the presence of HLA-B27 included those involved in *Salmonella *virulence, DNA replication, energy conversion and metabolism, and uptake and metabolism of nutrients.

**Table 1:** The leading genes that were differentially expressed between the two cell lines

Name	Genbank	Function and product	Fold changes**^α^**
*ssaN*	AAL20339	Secretion system apparatus	3.97
*ssaO*	AAL20340	Secretion system apparatus	2.23
*ssaR*	AAL20343	Secretion system apparatus	4.09
*ssaS*	AAL20344	Secretion system apparatus	3.25
*ssaV*	AAL20338	Secretion system apparatus	2.56
*sscA*	AAL20323	Secretion system apparatus	2.93
*sse I*	AAL19985	Gifsy-2 prophage; putative type III secreted protein	4.67
*sspH2*	AAL21143	Leucine-rich repeat protein, induced by the SPI-2 regulator ssrA/B	3.89
*dnaE*	AAL19195	DNA polymerase III, alpha subunit	2.68
*gyrA*	AAL21173	DNA gyrase, subunit A, type II topoisomerase	2.09
*gyrB*	AAL22694	DNA gyrase, subunit B, type II topoisomerase	2.39
*cyoC*	AAL19396	Cytochrome o ubiquinol oxidase subunit III	3.52
*cyoD*	AAL19395	Cytochrome o ubiquinol oxidase subunit IV	3.50
*nuoJ*	AAL21221	NADH dehydrogenase I chain J	2.19
*nuoK*	AAL21220	NADH dehydrogenase I chain K	2.42
*nuoM*	AAL21218	NADH dehydrogenase I chain M	2.39
*nuoN*	AAL21217	NADH dehydrogenase I chain N	2.27
*glnG*	AAL22844	Response regulator in two-component regulatory system with GlnL (EBP family)	2.88
*glnL*	AAL22845	Sensory kinase (phosphatase) in two-component regulatory system with GlnG	2.41
*glnA*	AAL22846	Glutamine synthetase	2.53
*gltB*	AAL22199	Glutamate synthase, large subunit	2.21
*gltD*	AAL22200	Glutamate synthase, small subunit	2.18
*aceF*	AAL19117	Pyruvate dehydrogenase, dihydrolipoyltransacetylase component	2.66
*fruA*	AAL21108	Sugar specific PTS system, fructose-specific transport	3.05
*fruF*	AAL21110	Phosphoenolpyruvate-dependent	2.67
		sugarphosphotransferase system, EIIA	
*pykF*	AAL20302	Pyruvate kinase I (formerly F), fructose stimulated	3.33
*pstC*	AAL22714	ABC superfamily (membrane), high affinity phosphate	0.48

^α ^FDR (*P-*value < 0.05) was set up during microarray data analysis.

### Up-regulation of SPI-2 genes may prevent damage by ROS

SPI-2 genes have been shown to make *Salmonella *capable of growing intracellularly by avoiding killing by reactive oxygen species (ROS) [38]. During *Salmonella *replication in macrophages, the innate defence systems of the host cells are triggered to limit the infection. ROS produced by phagocyte NADPH oxidase (*phox*) are the most effective anti-bacterial agents [39]. NADPH oxidase catalyzes the univalent reduction of molecular oxygen and produces superoxide, which has modest antibacterial activity but serves as a precursor for more toxic substrates, such as hydrogen peroxide and hydroxyl radicals [40]. Compared to those grown in HLA-A2 cells, *Salmonella *grown in HLA-B27 cells showed increased expression of SPI-2 genes including *ssaN, ssaO, ssaR, ssaS, ssaV, sscA, sseI and sspH2 *(Additional file 1: Supplemental Table S6-1). SPI-2 deficient mutants have impaired ability to grow in macrophages but are able to survive and are virulent within gp91*phox *knockout mice [39]. Patients with X-linked gp91*phox *mutations (chronic granulomatous disease, CGD) or gp91*phox *knockout mice that are unable to produce ROS are extremely susceptible to bacterial infection, including those caused by salmonellae [41,42]. Taken together, these findings indicated that SPI-2 is required for survival in the presence of ROS. The up-regulation of SPI-2 genes in HLA-B27 cells contributed to the increased intracellular growth and replication of *Salmonella*.

### Intracellular growth of *Salmonella *SPI-2 mutants affected by HLA-B27

**Figure 5:**
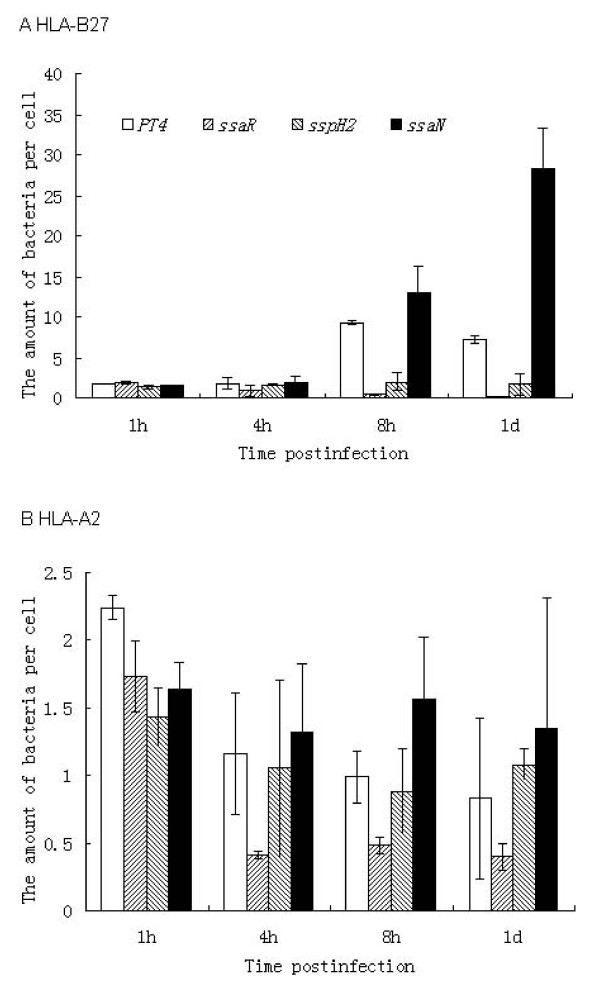
**Intracellular survival and replication of SPI-2 mutant *Salmonella *strains in U937 cells**. The cultured HLA-B27 or HLA-A2-transfected U937 cells were infected with complement-opsonized *S*. Enteritidis PT4 KS8822/88 wild type or mutants and incubation of the bacteria-infected cells was continued at 37°C for 1, 4, 8 or 24 h after infection. At the defined time points, the host cells were lysed and the number of bacteria per cell was reported as colony forming units (CFU). The numbers of bacteria in each HLA-B27 cell (A) or HLA-A2 cell (B) at the indicated time points are shown. Values are mean ± standard deviation from three independent experiments with duplicate samples. * *P *< 0.05, statistically significant differences between the wild type and mutant strains. Data were compared using Student's paired 2-tailed *t*-test.

To verify the significance of SPI-2 genes in *Salmonella *replication in HLA-B27 cells, three genes most markedly up-regulated in HLA-B27 cells - *ssaN*, *ssaR *and *sspH2 *- were deleted; *ssaN *and *ssaR *belong to the SPI-2 secretion apparatus and *sspH2 *is an effector gene. The intracellular growth of the mutants was compared with that of wild-type *S*. Enteritidis PT4 KS8822/88. Mutation of *ssaR *dramatically impaired intracellular *Salmonella *growth in HLA-B27 cells, decreasing it 19-fold at the 8 h and 33-fold at the 24 h time points (Figure. 5A). It has previously been shown that Δ*ssaR *mutants cannot grow intracellularly in murine macrophages or form the elongated *Salmonella*-induced filaments (Sifs) in epithelial cells [43] required for the formation of *Salmonella*-containing vacuoles (SCV) [44]. The formation and maintenance of SCV promotes the survival and proliferation of bacteria in macrophages [45] and the lack of formation of Sifs by *ssaR *mutants may account for the attenuation of this strain. The effector protein SspH2 is co-localized with vacuole-associated actin polymerization involved in the maintenance of SCV membrane integrity [46]. The *sspH2 *mutant was also attenuated more than 4-fold in HLA-B27 cells at both 8 and 24 hours after infection (Figure. 5A). Unexpectedly, the mutant carrying the *ssaN *deletion showed dramatically increased proliferation in HLA-B27 cells: 1.4-fold at the 8 h and 3.9-fold at the 24 h time points (Figure. 5A). The increased growth of the *ssaN *mutant in HLA-B27 cells might indicate that this mutant has good fitness in HLA-B27-positive U937 cells. The previous study by Eriksson et al [47] showed that the selection for *S*. Typhimurium mutants performed in murine macrophage-like J774-A.1 cells provided bacterial variants capable of a selective downregulation of nitric oxide (NO) expression. These variants showed increased host fitness. This is especially interesting, as in our model with murine L cells [30], iNOS activity and NO production were decreased in HLA-B27 positive cells, which correlated with increased *S*. Enteritidis growth. However, human U937 cells show very low iNOS activity and produce only small amounts of NO in general, and no difference between HLA-B27-positive and control cells was detected in this respect [48]. So the increased proliferation of the *ssaN *mutant in HLA-B27 cells, and no effect on growth in HLA-A2 cells (Figure 5B), perhaps indicates another specific and delicate relationship between *ssaN *and HLA-B27. These observations demonstrated a delicate balance between bacterial growth and pathogenesis.

### Regulation of bacterial DNA synthesis by HLA-B27

As *Salmonella *replicates more in HLA-B27-transfected cells than in HLA-A2 transfectants, it is not surprising that genes involved in DNA synthesis are up-regulated in HLA-B27 cells compared to HLA-A2 cells. DNA polymerase III (pol III) holoenzyme is a major replicase responsible for DNA synthesis [49]. It is a multiprotein complex containing over 10 distinct subunits. The α subunit encoded by *dnaE *is one component of the catalytic core (αεθ) of the pol III holoenzyme and mutations affecting the α unit result in reduced growth of *S. enteritica *serovar Typhimurium [50]. The expression of the gene *dnaE *was induced 2.7-fold in HLA-B27 cells compared to HLA-A2 cells at 8 h time point (Table 1). Other *dna *genes including *dnaA *encoding DNA replication initial protein, *dnaB *encoding putative replicative DNA helicases, and *dnaN *encoding β-subunit of DNA polymerase III were up-regulated 1.9-, 1.4-, and 2.0-fold, respectively. However, *dnaQ *encoding ε-subunit of DNA polymerase III was expressed at the similar level in two cell lines (see Additional file 1: Supplemental Table S1). The up-regulation of most *dna *genes in HLA-B27 cells compared to HLA-A2 cells might indicate more DNA replication in HLA-B27 cells. Two genes (*gyrA *and *gyrB*) required for the production of the heterotetramer-DNA gyrase were also up-regulated in HLA-B27 cells compared with the control cells. DNA gyrase is an essential enzyme for bacterial DNA synthesis; it introduces negative supercoils into DNA during replication [51]. The induction of *gyrA *and *gyrB *perhaps indicates that more DNA was synthesized by *Salmonella *resulting in increased bacterial proliferation in HLA-B27-transfected cells.

### *Salmonella *substrate and energy metabolism

Growth in a nutrient-limited environment may be echoed in changes in gene expression. Expression of genes involved in substrate and energy metabolism, including *nuoJKMN *encoding NADH dehydrogenase I chains (J, K, M and N components), *cyoCD *encoding cytochrome O ubiquinol oxidase subunits III and IV, and *aceF *encoding a putative dehydrogenase was up-regulated in HLA-B27 cells compared to HLA-A2 cells. This suggests that more energy was produced in HLA-B27 cells under aerobic conditions, which could be connected to the enhanced bacterial replication observed in HLA-B27 cells [52,53].

Up-regulation of amino acid metabolism by *Salmonella *growth in HLA-B27 cells compared to HLA-A2 cells was evident from the induction of *gltB*, *gltD *and *glnA *encoding glutamate synthase/glutamine synthetase, and *glnGL *encoding a two-component regulator of glutamine synthetase [54]. Glutamate is an important precursor of other amino acids and of pyrimidine and purine synthesis in bacteria [55]. Moreover, sugar catabolism genes such as *aceF*, *fruA*, *fruF *and *pykF *were elevated in HLA-B27 cells. Collectively, these data suggest that HLA-B27 affects intracellular substrate metabolism and energy production by *Salmonella *during infection of U937 cells, probably due to the increased bacterial proliferation in these cells.

### HLA-B27 modified ionic transport systems

Magnesium is involved in the stabilisation of the cellular membrane and functions as a coenzyme. MgtA and MgtB are two inducible transporters, and high up-regulation of *mgtB *and *mgtA *in both HLA-B27 and HLA-A2 macrophages might suggest that Mg^2+ ^is extremely limited within the SCV of infected U937 cells [56,57]. From 1 to 8 h after infection, the degree to which these two genes were up-regulated was increased in both cell lines, indicating that the amounts of magnesium decreased during the course of infection. MgtC is also involved in Mg^2+ ^transport in the intracellular survival of *Salmonella *and other bacteria in macrophages [58,59]. The two genes *mgtC *and *mgtB *are located in one operon, *mgtCB*, which belongs to SPI-3 on the *Salmonella *chromosome [58]. Our data showed that both *mgtC *and *mgtB *were less induced in HLA-B27-positive cells than in HLA-B27-negative cells, suggesting that HLA-B27 modulates the acquisition of Mg^2+ ^in macrophages.

Phosphate availability is limited in macrophage vacuoles [60]. These observations are supported by our microarray data showing that the *phoBR *regulon, the *pstC *and *pstS *genes responsible for phosphate uptake and/or transport, were up-regulated during intracellular growth. The genes were induced to similar levels in both cell lines at 2 h after infection but the expression of *pstC *was down-regulated in HLA-B27 cells compared to HLA-A2 cells at 8 h postinfection. Taken together, these results suggest that HLA-B27 affects the intracellular ionic status of the SCV.

## Conclusions

We have elucidated the global gene expression profile of *S*. Enteritidis PT4 KS8822/88 during intracellular growth in human monocyte/macrophages for the first time. The gene expression profile shown by *S*. Enteritidis during intracellular growth is very different from that exhibited in LB broth. However, it is similar to the patterns of gene expression reported for *S*. Typhimurium bacterial intracellular growth in macrophages [52]. Approximately one quarter of *S*. Enteritidis genes showed up- or down-regulation in host cells compared to LB broth. Among the genes up-regulated intracellularly were SPI-2 virulence genes, nutrient acquisition system genes, and ionic uptake and/or synthesis genes. Similar differences in intracellular *S*. Typhimurium gene expression were also seen between macrophages and medium *in vitro *[52]. SPI-2 genes are broadly induced to evade immune responses by host cells. Acquisition and transport system genes were elevated to take up the necessary elements. Among the down-regulated genes, some were related to central metabolism, such as the tricarboxylic acid cycle and oxidative phosphorylation, indicating that the level of oxygen is extremely low in the SCV intracellular environment, which seems contrary to the observation of intracellular *S*. Typhimurium growth [52]. Also, *Salmonella *metabolism and energy production were slower intracellularly than during growth in LB medium. These changes reflect the intracellular environment that the bacteria encounter during infection of macrophages [61].

The survival and proliferation of pathogens within macrophages is critical for establishing systemic infection, and this process is related to bacterial gene expression [62,63]. The expression of HLA-B27 in the U937 cells appeared to generate a favourable environment for intracellular *Salmonella *growth compared with HLA-A2-transfected cells, resulting in more bacteria in HLA-B27-transfected cells. The increased ability of *Salmonella *to survive is probably due to the altered gene expression. The expression of 118 genes at 8 h after infection differed significantly between HLA-B27 cells and HLA-A2 cells, suggesting that HLA-B27 modified *Salmonella *gene expression and/or affected the intracellular bacterial growth environment during the infection of macrophages. Expression of SPI-2 genes, which play a key role in the increased persistence of *Salmonella *in macrophages, was up-regulated in HLA-B27 cells compared to HLA-A2 cells. DNA replication, energy production and nutrient metabolism were also increased, suggesting that more bacterial physiological activities occurred in HLA-B27 cells. This is consistent with the increased replication and survival of *Salmonella *in HLA-B27-transfected macrophages. Interestingly, expression of many SPI-2 genes, energy production and nutrient metabolism are also increased in epithelial cells where *Salmonella *replicates intracellularly [64], whereas e.g. Cyo and Cyd terminal oxidases, which use oxygen exclusively as terminal acceptor, are strongly downregulated in *Salmonella *inhabiting restrictive cells, fibroblasts [65] and HLA-A2 cells (Additional file 1: Supplemental Table S2-2 and S4-2).

Mutagenesis of the SPI-2 genes demonstrated the crucial role of SPI-2 in the survival and replication of *Salmonella *in HLA-B27 cells, as has been seen in other macrophages [18,19]. The intracellular replication and survival of the strains bearing deletion of either *ssaR *or *sspH2 *was impaired in comparison with wild-type PT4 (Figure. 5A). Both proteins participate in the maintenance of the SCV membrane, which is important for the survival of *Salmonella *in infected macrophages. Increased growth of the *ssaN *mutant in HLA-B27-positive cells might indicate a special connection between this mutant and HLA-B27-positive cells, and may even be relevant to the pathogenesis of HLA-B27-associated ReA.

HLA-B27 modifies the host cell's signalling pathways during *Salmonella *infection. Our group recently demonstrated that mitogen activated protein kinase (MAPK) p38 is involved in *Salmonella *replication in U937 cells, and that the p38 MAPK pathway is deregulated in HLA-B27-transfected cells [33]. *Salmonella *uses type III secretion systems (T3SS) encoded in SPI-1 or -2 to deliver virulence proteins into host cells, which subsequently interfere with the host cell's signalling pathways [15,66,67]. Further experiments will be required to determine how *Salmonella *genes, particularly SPI-2 genes, affect intracellular signalling pathways and cause increased replication of bacteria in HLA-B27 cells. Nevertheless, these findings give new insights into the disturbed microbe-host interaction in HLA-B27-positive cells.

## Methods

### Cell lines and transfections

The human monocytic U937 cell line was obtained from ATCC (Rockville, USA). The full-length 6-kb genomic clone of human HLA-B*2705 DNA [68] in the vector pUC19 and the full-length 5.1-kb genomic clone of human HLA-A2 DNA [69] in the vector pUC9 were kind gifts from Dr. Joel D. Taurog. For transfection, U937 cells were suspended in RPMI 1640 containing 1.8 mM L-glutamine and 1 mM sodium pyruvate (Gibco, Paisley, Scotland), and were then cotransfected by electroporation with the vectors carrying either HLA-B*2705 or HLA-A2 DNA with the plasmid pSV2neo (to provide resistance to geneticin [G-418]) (CalBiochem, Darmstadt, Germany). Stable transfectants were selected with 0.5 mg/ml geneticin and the expression of transfected HLA-B27 or -A2 molecules on the surface of U937 cells was examined by immunofluorescence (IF) and flow cytometry as described previously [34]. The transfected cell lines were maintained in RPMI 1640 supplemented with 10% heat-inactivated fetal calf serum (FCS; PAA laboratories, Linz, Austria), 1.8 mM L-glutamine and 50 μg/ml gentamicin (Gm) (both from Biological Institutes, Kibbutz Beit Herennek, Israel) at 37°C in a humidified atmosphere of 5% CO_2_/95% air. Cell cultures were tested to confirm freedom from mycoplasma contamination.

### Bacterial strains, plasmids and culture conditions

Bacterial strains and plasmids used in this study are listed in Table 2. The wild type *S*. Enteritidis strain was originally isolated from the stool of a patient with *Salmonella*-triggered ReA [34], and was typed as phage type 4 (PT4) and named *S*. Enteritidis PT4 KS8822/88. The *S*. Enteritidis and *Escherichia coli *strains were routinely grown in Luria-Bertani (LB) broth at 37°C. Bacteria carrying a temperature-sensitive plasmid (pKOBEGA) were grown at 30°C. Media were supplemented with 100 μg/ml ampicillin (Amp) (Sigma) and 30 μg/ml kanamycin (Km) (Sigma) as required.

**Table 2:** Bacterial strains and plasmids used in this study

Strains and plasmids	Characteristics	Source/reference
Strains		
*S*. Enteritidis PT4	Wild-type clinical isolate	34^α^
PT4 (pKOBEGA)	PT4 containing helper plasmid pKOBEGA, Amp^R^	This study
PT4Δ*ssaN*	PT4 Δ*ssaN*::Km, in-frame deletion, Km^R^	This study
PT4Δ*ssaR*	PT4 Δ*ssaR*::Km, in-frame deletion, Km^R^	This study
PT4Δ*sspH2*	PT4 Δ*sspH2*::Km, in-frame deletion, Km^R^	This study
*E. coli *MC4100	Used as template for amplication of kanamycin resistance gene	Gift from J. M. Ghigo
Plasmid pKOBEGA	Vector for recombination experiments, Amp^R^	Gift from J. M.Ghigo

^α ^: *S*. Enteritidis strain was typed as PT4 and named as the strain KS8822/88 in this study.

### Cell infection model

For infection, the U937 cells were diluted to 1.0 × 10^6 ^cells/ml and then seeded in tissue culture flasks (75 cm^2^) or 24-well plates (Greiner, Germany). Prior to bacterial infection, the cells were cultured with phorbol myristate acetate (PMA; Sigma) for 24 h to differentiate them toward more mature macrophage-like cells. Two hours before infection, the adherent cells were washed with Hank's balanced salt solution (HBSS) and then overlaid with prewarmed RPMI 1640 supplemented with 10% human AB serum (Finnish Red Cross, Helsinki, Finland). The cells were then co-cultured with *Salmonella *at a multiplicity of infection (MOI) around 50:1. After 2 h of infection, the cells were washed three times with HBSS to remove non-adherent bacteria and overlaid with fresh RPMI 1640 containing 50 μg/ml Gm to kill extracellular bacteria. The *Salmonella*-infected cells were then incubated at 37°C for 1, 4, 8 or 24 hours as indicated. To determine the number of living intracellular bacteria, the infected cells were scraped using a cell scraper and the amount of living host cells was counted under a microscope after staining with Trypan blue. Host cells were lysed with 1% Triton X-100 in 1 × phosphate-buffered saline (PBS) at room temperature for 5-10 min, 20 μl of the lysate in ten-fold serial dilutions in PBS were added to LB agar plates, and the numbers of bacteria were reported as CFU (colony forming units).

### RNA extraction

Two methods were used to extract bacterial RNA from infected cells. At 1 or 8 h post infection, the intracellular bacteria were recovered from infected macrophages and lysed with 1% Triton X-100 [34]. The lysate was first centrifuged at 250 × g at 4°C to remove pieces of broken host cells and then the supernatant was centrifuged at 12,000 × g for 5 min to precipitate bacterial cells. The approach developed for recovering bacteria from infected cells ensured the minimum contamination of extracted bacterial RNA with eukaryotic RNA. Otherwise, infected macrophages were lysed in 0.1% SDS, 1% acidic phenol, 19% ethanol in water on ice for 30 min [60] and similar results were obtained. Total bacterial RNA was extracted and purified using the Promega SV total RNA purification kit according to the manufacturer's instructions (Promega). Control RNAs from bacteria grown in LB broth to mid-logarithmic phase and from eukaryotic cells were isolated using the same RNA purification kit. RNA integrity was monitored by agarose gel electrophoresis and using the Agilent 2100 system (Agilent). The concentration and purity of RNA was measured by spectrophotometry Bio-RAD SmartSpee™3000 (Bio-Rad).

### Microarray hybridisation

The 'SALSA' *Salmonella *serovar microarray was developed with 5080 *Salmonella *genes from different serovars of *Salmonella *including 196 *S*. Enteritidis PT4 specific genes. More details are available at http://www.ifr.bbsrc.ac.uk/safety/microarrays/#protocols. A type II experiment design was used for hybridisation with labelled bacterial genomic DNA as a reference channel in each experiment [70]. The protocol for hybridisation was as previously described [52]. Briefly, total bacterial RNA (5 μg) from *Salmonella *grown in host cells or in LB broth was converted into cDNA using Superscript II reverse transcriptase and random primer as recommended by the manufacturer. The cDNA produced from the reverse transcription reaction and genomic DNA was subsequently fluorescently labelled using Cy5 or Cy3 dyes by random priming, with increasing labelling efficiency using Klenow enzyme. Labelled cDNA and genomic DNA were hybridized with the microarray slides overnight at 65°C. After hybridization, the slides were carefully washed, dried and scanned. All hybridizations were performed with at least three biological replicates.

### Microarray data analysis

Fluorescent intensity data from each array were collected using a GenePix 4000A scanner (Axon Instruments). To compensate for unequal dye incorporation, data were centred by bringing the median natural logarithm of the ratios for each group of spots printed by the same pin to zero. The data were analyzed using GENE-SPRING™6 software (Silicon Genetics). The significance of the centred data at *P *= 0.05 was determined using a parametric-based statistical test, adjusting the individual *P*-value by the Benjamini and Hochberg false discovery rate multiple test correction.

### Microarray data accession number

The supporting microarray data have been deposited in the Array Express database http://www.ebi.ac.uk/arrayexpress with accession number E-MEXP-1438.

### Quantitative real time RT-PCR

Primer3 software http://frodo.wi.mit.edu[71] was used to design the primers for quantitative real time RT-PCR and purchased from Sigma (Table 3). The primers were designed to have similar melting temperatures and lengths of PCR products. About 1 μg of total RNA from the bacteria after 2 or 8 h infection of U937 cells or growth in LB medium was reverse-transcribed to 1^st ^strand cDNA using AMV reverse transcriptase and the random primer provided in the kit following the manufacturer's instructions (1^st ^Strand cDNA Synthesis Kit for RT-PCR (AMV)^+^, Roche). The resultant cDNA was used as a template during PCR, which was performed on a Roche LightCycler instrument and using the LightCycler FastStart DNA Master SYBR green I kit (Roche). The protocol for the PCR reaction was an initial denaturation at 95°C for 10 min, followed by 40 cycles of denaturation at 95°C for 5 s, annealing at 56-64°C for 5 s and extension at 72°C for 10 s. For certain pairs of primers the annealing temperatures might be changed to optimize the yield of products. The amplified specific products were examined by melting temperature curves and agarose gel electrophoresis. Relative gene expression was quantified using a standard curve method plotted from ten-fold serial dilutions of known quantities of cDNA samples. Each standard curve for each gene was then used to transform threshold cycle (Ct) to the relative amount of unknown cDNA of the gene. The analysis was based on the Ct values of the specific genes, with *rfaH *as a normalizer. The amount of the specific gene was divided by the amount of *rfaH *in the same cDNA preparation to calculate the normalized value. Relative gene expression was presented as ratios of the normalized values of cDNA from bacterial growth in U937 cells to the growth in LB broth. All experiments were performed in triplicate using independent RNA preparations (biological replicates).

**Table 3:** Primers used in the real-time RT-PCR in this study

Genes	Primers	Sequences
*mgtC*	mgtC-F	5'-GTCTCTGGTATTGGCTTTCTGG-3'
	mgtC-R	5'-TTGGCACAAAGAATAATGATCG-3'
*rpoE*	rpoE-K	5'-GACGCGATTGAAGCAGAAA-3'
	rpoE-R	5'-CCAGCTCCCGTAAGGTGAT-3'
*ssaG*	ssaG-F	5'-TTAGTGGATATGCTCTCCCACA-3'
	ssaG-R	5'-TCATTTTGATCAGTGAACTTTCG-3'
*fliC*	fliC-F	5'-AGCCTGTCGCTGTTGACC-3'
	fliC-R	5'-CGCTGCAGGTTGTGGTTG-3'
*fumC*	fumC-F	5'-CGGTATGGAACGCAAAGTG-3'
	fumC-R	5'-CTCCTGGCCTAAGGTGAGC-3'
*nuoA*	nuoA-F	5'-TGCTGCCTGATGCTGGTA-3'
	unoA-R	5'-CGCTTTCGCGGATAGAAG-3'
*rfaH*	rfaH-F	5'-ACTTCAGCGTGCTCAGGAA-3'
	rfaH-R	5'-GCGTGGCGTTGATTGTAGT-3'

### DNA manipulations and preparation of electrocompetent cells

Plasmid DNA from *E. coli *was purified with a Quantum Prep plasmid miniprep kit (Bio-Rad). Plasmids were transformed into the strain *S*. Enteritidis PT4 KS8822/88 by electroporation. Transformants carrying the Red helper plasmid were made electrocompetent as described previously [72] with some modifications. Cells were grown overnight in LB broth Amp at 30°C and then 1 ml of overnight cultures were used to inoculate 100 ml of LB broth Amp and incubation was continued to an OD_600 _of 0.15-0.2. L-arabinose (Sigma) was then added to a final concentration of 10 mM and incubation was continued until the OD_600 _reached 0.7. The suspension was cooled on ice for 20 min, and the cells were made electrocompetent by washing twice with the same volume of ice-cold water and then once with 40 ml of ice-cold 10% glycerol. The cells were finally resuspended in 1 ml of ice-cold 10% glycerol, then divided into 50 μl working aliquots and kept at -70°C for up to two months. Total genomic DNA was isolated according to the manufacturer's instructions (High Pure PCR template Preparation kit, Roche).

### Construction of gene mutants by the λ Red mutagenesis method

*Salmonella *genes were disrupted by the method described previously [72,73]. Briefly, purified plasmid pKOBEGA was introduced into *Salmonella *by electroporation, and transformants were selected on LB agar Amp after incubation for 24 h at 30°C. The λ Red helper plasmid pKOBEGA is a low-copy-number plasmid that contains an ampicillin resistance gene, a temperature-sensitive origin of replication and the Red system, including three genes expressing the Exo, Bet and Gam functions of phage λ, which helps allelic exchanges between linear DNA and the corresponding region on the chromosome [74]. The *Salmonella *strains carrying the λ Red helper plasmid were made electrocompetent as described above. PCR linear DNA fragments were generated with specific hybrid primers and high-fidelity thermophilic DNA polymerase (Dynazyme Ext; Finnzymes). The primer pairs shown in Table 4 were used. PCR products were purified with a QIAquick PCR Purification kit (QIAGEN) and 5 μg of purified product (5-10 μl) was electroporated (25 μF, 200 Ω, 2.5 kV) into 50 μl of eletrocompetent cells according to the manufacturer's instructions (Bio-Rad), then 950 μl of LB broth was added to the shocked cells and the solution was transferred into clear eppendorf tubes and incubated for 2 h at 37°C, and then spread on to LB agar Km to select Km transformants after overnight growth at 37°C. Selected antibiotic resistance colonies were then grown on LB broth Km at 43°C for 24 h and then spread on LB agar Km or Amp at 37°C to check the loss of the helper plasmid. The mutated genes were confirmed by PCR using the primers shown in Table 4, which are external to the site of mutagenesis with the internal deletion replaced by the kanamycin cassette.

**Table 4:** Primers used in the gene mutagenesis in this study

Genes	Primers	Sequences
ssaN	ssaN-Km-F	5'-CTTTGCTATCTCCTTTTACGAGTACAATCGGGCTTCACTGC
		GGGCAGCAAGTGATGGCCTAAAGCCACGTTGTGTCTCAA-3'^α^
	ssaN-Km-R	5'-CTTCAGACAGTGTAAAATCGATGAATTCGCGGACTTCTCGT
		CCACGTTCACCAATTAACAGCGCTGAGGTCTGCCTCGTG-3'^α^
	ssaN-F	5'-CGTTGTTAAATGCGTGGTTG-3'
	ssaN-R	5'-CCCATTCCCGTACGTTCTAA-3'
ssaR	ssaR-Km-F	5'-GCGCTTGTACTTTCCTTATTCATTATGGGGCCGACGCTATT
		AGCTGTAAAAGAGCGCTGGAAAGCCACGTTGTGTCTCAA-3'^α^
	ssaR-Km-R	5'-ATTAATATGAGCAAAGAATCAGGTTTTATCTTTCTTTTTAT
		GTTCTTCAGGCCAGGTTCGGCGCTGAGGTCTGCCTCGTG-3'^α^
	ssaR-F	5'-TATCGCACTGTATGGCCTTG-3'
	ssaR-R	5'-ACCGCCTGCCAGTAAAAATA-3'
sspH2	sspH2-Km-F	5'-CTTTATGAAGTTTTCCGTCTCACTCAGTCTGTCCAGGAAGA
		GGCTGAATGCGTCGGCGTTAAAGCCACGTTGTGTCTCAA-3'^α^
	sspH2-Km-R	5'-ACACTGGTTATTCCTGATAATAATCTGACCAGCCTGCCGGC
		GCTGCCGCCAGAACTGCGGGCGCTGAGGTTGTGTCTCAA-3'^α^
	sspH2-F	5'-TCATCTTCAGCCAGTTGTGC-3'
	sspH2-R	5'-GTAATCGCCGCATTTATCGT-3'

^α^: 80-nucleotide (nt)-long primers including 60 nt homology extensions complementary to the targeted regions of the genes and 20 nt priming sequences (underlined) for the synthesis of the kanamycin resistance cassette gene from *E. coli *MC4100 *ybeW*::Km.

## Authors' contributions

SCG designed and carried out the experiments, and wrote the manuscript. VD performed the microarray tests and analyzed the data. QSH designed the experiments and analyzed the data. JCDH conceived and designed the experiments. KG conceived and designed the experiments, and wrote the manuscript. All authors reviewed the manuscript.

## Supplementary Material

Additional file 1 **Supplemental Tables S1- 6 - Global gene expression of *S*. Enteritidis PT4 in HLA-B27- or HLA-A2-transfected cells using microarray assay**. The Supplemental Tables S1-6 contain gene expression profiles of *S*. Enteritidis PT4 strain during infection of U937 cells (Table S1), genes up- or down-regulated more than two-fold in HLA-A2-transfected U937 cells compared to LB culture at 2 h after infection (Tables S2-1, -2), in HLA-B27-transfected cells compared to LB culture at 2 h after infection (Table S3-1,-2), in HLA-A2-transfected U937 cells compared to LB culture at 8 h after infection (Tables S4-1, -2), in HLA-B27-transfected cells compared to LB culture at 8 h after infection (Table S5-1,-2) and in HLA-B27-transfected cells compared to HLA-A2-transfected cells at 8 h after infection (Tables S6-1, -2). Click here for file

Additional file 2 **Supplemental Tables S7 and S8 - The correlation analysis between PT-PCR and array data and the expression of the gene *rfaH *in different conditions**. The Supplemental Tables S7 and S8 contain the correlation analysis between PT-PCR and array data (Table S7) and the expression of the gene *rfaH *in different conditions (Table S8). Click here for file
